# Benign cephalic histiocytosis in a 2‐year‐old boy with an inconspicuous clinical presentation at onset

**DOI:** 10.1002/ccr3.8043

**Published:** 2023-10-10

**Authors:** Sandra Osorio, Lucie Harpain, Karin Jahn‐Bassler, Adrian Tanew, Sonja Radakovic

**Affiliations:** ^1^ Department of Dermatology Medical University of Vienna Vienna Austria; ^2^ Private Practice Vienna Austria

**Keywords:** benign cephalic histiocytosis, facial dermatoses, histiocytosis, infant

## Abstract

We report on a 2‐year‐old boy whose initially inconspicuous skin lesions later on evolved into a typical clinical presentation of benign cephalic histiocytosis (BCH). The diagnosis of BCH can often be made on clinical grounds without the need for an extensive diagnostic work‐up. Given the benign and self‐limited course of the disease treatment is not required and the clinical management can be limited to a watchful waiting approach.

## Graphical abstract

A case with typical clinical presentation of benign cephalic histiocytosis.

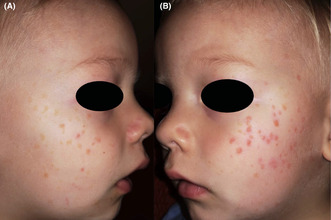

## 1 INTRODUCTION

In 1971 Gianotti et al. first described benign cephalic histiocytosis (BCH) and up to the present approximately 70 cases have been reported in the literature.^1^, ^2^ In 2016 the Working Group of the Histiocyte Society published a revised classification of histiocytoses and neoplasms of the macrophage‐dendritic cell lineages based on clinical, radiographic, pathological, phenotypic, genetic, and/or molecular features. According to this classification BCH as a part of the xanthogranuloma family falls into group C together with juvenile xanthogranuloma (JXG), adult xanthogranuloma (AXG), solitary reticulohistiocytoma (SRH), generalized eruptive histiocytosis (GEH) and progressive nodular histiocytosis PNH.^3^, ^4^ Clinically and histologically BCH resembles JXG and GEH. Skin lesions are characterized by erythematous, yellowish to brownish macules and papules, which are mostly located on head and neck. The etiology of BCH is as yet unresolved.

## 2 CASE REPORT

**FIGURE 1:**
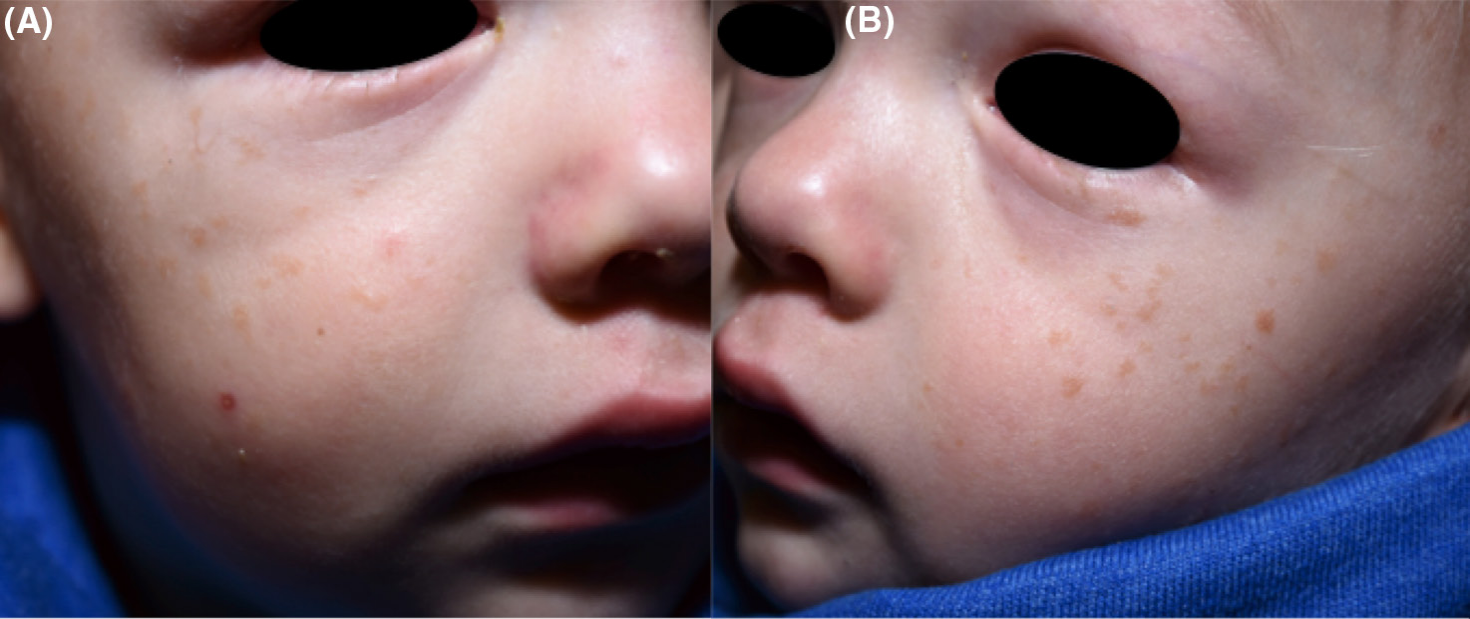
(A, B) Light brown macules and flat papules at first presentation.

**FIGURE 2:**
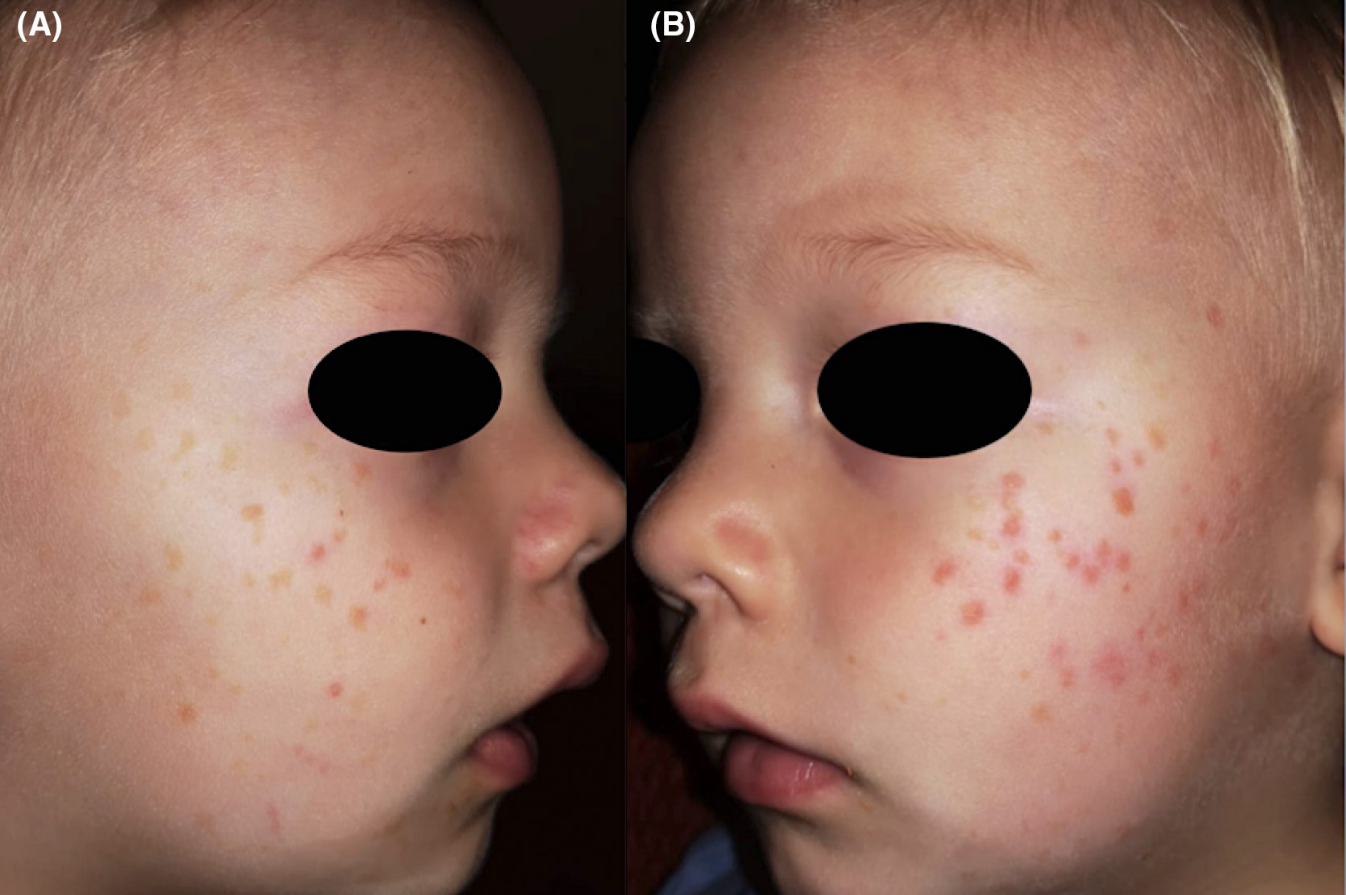
(A, B) Reddish brown papules 5 months after the initial presentation.

A 2‐year‐old boy with a fair complexion presented with multiple, light brown macules, evenly distributed on the cheeks and the forehead (Figure 1A,B). The family history was negative for skin diseases. According to the mother, small macular lesions had appeared at the age of 9 months on the left temple without any preceding inflammation. Subsequently, new lesions developed, particularly after the summer. Differential diagnoses included juvenile warts, freckles, and urticaria pigmentosa. The Darier sign was negative. As the lesions were restricted to the face and increased after environmental sun exposure also a diagnosis of xeroderma pigmentosum (XP) was considered. However, genetic testing for a mutation in the *ERCC*3 gene was negative. Five months later the number of lesions had increased and the macules had evolved into monomorphic flat reddish brown papules. (Figure 2A,B) The clinical presentation now indicated a diagnosis of BCH. Confirmation of the clinical diagnosis by histopathology was not possible, as the mother did not consent to a biopsy from the face, where at that time the lesions were exclusively located. A complete blood count, comprehensive blood chemistry, and an abdominal ultrasound were all unrevealing. In light of the benign nature of BCH and its propensity for spontaneous resolution a watchful waiting approach without any treatment was chosen.

**FIGURE 3:**
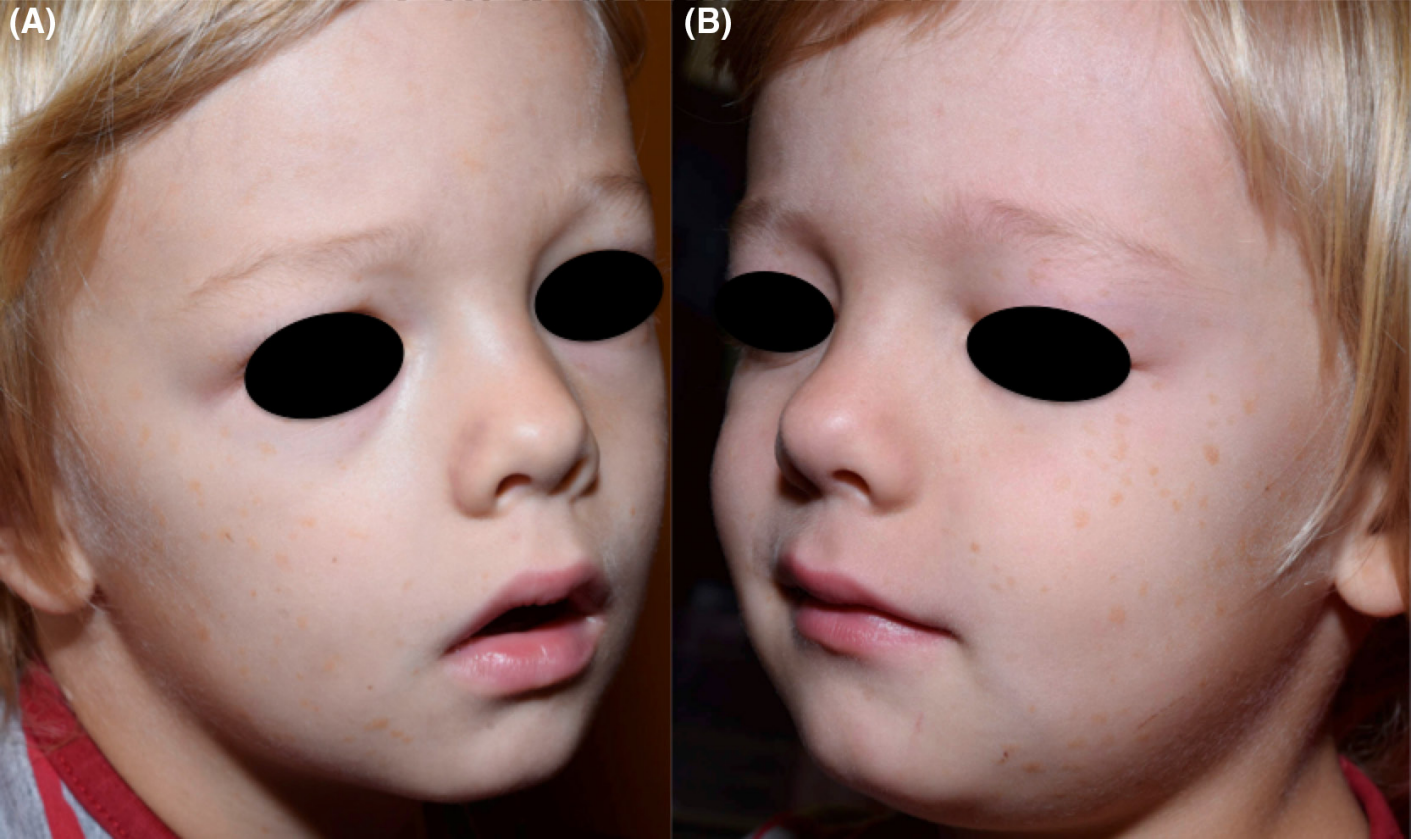
(A, B) Regression to light brown macules and flat papules after 2 years.

At follow‐up examinations 9 and 16 months later a few new lesions had developed in the pelvic area and on the dorsal forearms and none of the preexisting lesions had regressed. Two and a half year later part of the extracephalic lesions had completely disappeared and the lesions on the head were fading (Figure 3A,B).

## 3 DISCUSSION

The diagnosis of BCH is made on clinical grounds. Diagnostic criteria are the manifestation in early childhood (average 15 months), the typical clinical morphology and the predominant distribution in the head and neck region. However, contrary to its original designation as BCH, several recent case reports have shown that extracephalic skin involvement, particularly on the upper trunk, is also commonly found.^2^, ^5^, ^6^

BCH is a benign disease without systemic involvement that runs a self‐limited course. The lesions usually resolve within 50 months with occasional mild atrophy and hyperpigmentation.^5^, ^6^, ^7^ Recently, improvement of skin lesions has been reported in a 5‐year‐old boy with extensive, progressive BCH involving the face, trunk, and extremities after twice‐daily treatment with topical 1% rapamycin.^8^

Differential diagnosis primarily includes the two prevailing types of NLCH, JXG, and GEH. JXG is characterized by many small pink to red–brown, dome shaped papules scattered on the upper part of the body that rapidly become yellow (micronodular JXG) or by a few nodular lesions (large nodular JXG). JXG mostly occurs in infants or young adults and may be associated in approximately 5% of pediatric cases with extracutaneous involvement especially of the eyes, liver, and lung. An association with neurofibromatosis I, juvenile chronic myelomonocytic leukemia, and LHC has also been described. GEH typically affects adults and presents as recurrent crops of generalized numerous red to brown papules with a widespread distribution. GEH can be associated with hematological neoplasia. Clinical and histopathological distinction between an early phase of small nodular JXG or GEH and BCH may occasionally be difficult or even impossible. Thus it has been proposed that these diseases might be variants of a single clinical entity.^4^, ^9^, ^10^, ^11^

In the present case the diagnosis of BCH was based on clinical criteria that included a disease onset in early childhood (on average 15 months), the typical clinical morphology of the lesions and their predominant distribution in the head and neck region. A skin biopsy is generally not required and should be reserved for doubtful cases to allow for differentiation from other types of cutaneous histiocytoses.

In summary, our case brings to attention the clinical characteristics of BCH and points to the fact that BCH may not be confined to the cephalic region. We also discuss the distinction of BCH from other types of NLCH and underline the benign course of this disease, which does not require an extensive diagnostic work‐up but only a regular clinical monitoring.

## AUTHOR CONTRIBUTIONS

**Sandra Osorio:** Conceptualization; investigation; validation; writing – original draft; writing – review and editing. **Lucie Harpain:** Investigation; validation; visualization; writing – review and editing. **Karin Jahn‐Bassler:** Investigation; validation; writing – review and editing. **Adrian Tanew:** Funding acquisition; project administration; supervision; validation; writing – review and editing. **Sonja Radakovic:** Conceptualization; funding acquisition; investigation; project administration; supervision; validation; visualization; writing – original draft; writing – review and editing.

## FUNDING INFORMATION

None.

## CONSENT STATEMENT

All patients in this manuscript have given written informed consent for participation in the study and the use of their de‐identified, anonymized, aggregated data, and their case details (including photographs) for publication.

## Data Availability

Data are available on request from the corresponding author.
